# Entropic Movement Complexity Reflects Subjective Creativity Rankings of Visualized Hand Motion Trajectories

**DOI:** 10.3389/fpsyg.2015.01879

**Published:** 2015-12-17

**Authors:** Zhen Peng, Daniel A. Braun

**Affiliations:** ^1^Max Planck Institute for Biological CyberneticsTübingen, Germany; ^2^Max Planck Institute for Intelligent SystemsTübingen, Germany; ^3^International Max Planck Research School for Cognitive and Systems NeuroscienceTübingen, Germany

**Keywords:** creativity, motion complexity, Gaussian processes, probabilistic movement primitives, Lempel-Ziv complexity

## Abstract

In a previous study we have shown that human motion trajectories can be characterized by translating continuous trajectories into symbol sequences with well-defined complexity measures. Here we test the hypothesis that the motion complexity individuals generate in their movements might be correlated to the degree of creativity assigned by a human observer to the visualized motion trajectories. We asked participants to generate 55 novel hand movement patterns in virtual reality, where each pattern had to be repeated 10 times in a row to ensure reproducibility. This allowed us to estimate a probability distribution over trajectories for each pattern. We assessed motion complexity not only by the previously proposed complexity measures on symbolic sequences, but we also propose two novel complexity measures that can be directly applied to the distributions over trajectories based on the frameworks of Gaussian Processes and Probabilistic Movement Primitives. In contrast to previous studies, these new methods allow computing complexities of individual motion patterns from very few sample trajectories. We compared the different complexity measures to how a group of independent jurors rank ordered the recorded motion trajectories according to their personal creativity judgment. We found three entropic complexity measures that correlate significantly with human creativity judgment and discuss differences between the measures. We also test whether these complexity measures correlate with individual creativity in divergent thinking tasks, but do not find any consistent correlation. Our results suggest that entropic complexity measures of hand motion may reveal domain-specific individual differences in kinesthetic creativity.

## Introduction

Creativity is a hallmark of human behavior and the motor for innovation both in individuals and in society. Machines typically lack this ability, as they only execute commands they are programmed for, usually under very specified conditions. Consequently, there is a growing interest in investigating how creativity could be modeled in a quantitative way and automatized (Boden, 1998). Practical examples for the study of computational creativity in artificial systems include linguistic processing (Binsted et al., 1997), composition of music (Gibson and Byrne, 1991) and visual art (Romero and Machado, 2008). An overarching theoretical conceptualization has been proposed by Schmidhuber suggesting that creativity can be understood in the context of intrinsic rewards that come from finding novel patterns or regularities allowing for improved data compression of previous observations (Schmidhuber, 2010, 2012). According to this theory there is a fundamental link between creativity and informational complexity.

In this study we test experimentally for the link between creativity and informational complexity in the domain of human hand movements. Human creativity expresses itself in many domains and across different sense modalities ranging from visual, to auditory and kinesthetic, as evidenced for example in the visual arts, in music and in dance. However, this does not necessarily mean that there is a single monolithic cognitive process underlying creativity and, in fact, it has been stipulated that creativity could be highly domain-specific (Baer, 1998; Plucker, 1998; Baer, 2008). What makes human hand movements particularly amenable to a quantitative study of a domain-specific creativity is that we could show previously that informational complexity can be used to characterize the complexity of different types of hand motion (Peng et al., 2014). These previous measures require that we translate motion trajectories into symbol sequences consisting of transitions between the four cardinal movement directions. Due to the limited number of symbolic transitions in short movements, however, we were previously unable to estimate the informational complexity of particular motion patterns. In this study we adopt two modern machine learning approaches that are based on Gaussian Processes and Probabilistic Movement Primitives both of which allow representing probability distributions over trajectories. This way we cannot only evaluate the complexity of particular motion patterns, but also evaluate the empirical adequacy of Schmidhuber's theory by relating the informational complexity of motion patterns to measures of kinesthetic creativity.

In general, creativity can be assessed in different ways. Most researchers distinguish between creative potential and creative achievement (Eysenck, 1995; Jauk et al., 2013), where the former refers to a basic individual ability that follows a normal distribution in the population, whereas the latter refers to actual real-life accomplishments like publishing a book, composing a piece of music or making a scientific discovery. While creative achievement is usually measured by auto-biographical self-reports like the Creative Achievement Questionnaire (Carson et al., 2005), creative potential is usually measured by psychometric tests. Guildford was one of the first to suggest that creative potential could be assessed by means of testing the ability of divergent thinking when solving open problems that allow for a variety of solutions (Guilford, 1967). Ever since, several attempts have been made to develop psychometric measures of a “creativity quotient” of an individual similar to the intelligence quotient (IQ). Well-known tests include the Guildford tests (Guilford, 1967), the Wallach and Kogan tests (Wallach and Kogan, 1965) and the Torrance Test of Creative Thinking (Torrance, 1966, 1974). The Torrance Test originally involved simple tests of divergent thinking and other problem-solving skills based on fluency, flexibility, originality and elaboration. It is the most widely used test, for example to assess the giftedness of school kids, and has been renormed four times since its inception (Kim, 2006).

Instead of assessing the creative potential of a person by psychometric measurement, it has also been proposed to assess the creativity of artifacts created by the person (Hennessey and Amabile, 2010). Such assessments usually follow the Consensual Assessment Technique (CAT; Amabile, 1982). Unlike other measures of creativity, which are often based on a particular theory of creativity, the CAT is based on the idea that the best measure of the creativity of a work of art, a scientific proposal, or any other artifact is the combined assessment of experts in the same domain (Baer and McKool, 2009). This approach enjoys wide popularity, as it is theory-independent, relatively simple to implement and usually achieves high levels of inter-rater agreement (Hennessey and Amabile, 2010). In our study we test the hypothesis that informational complexity of human hand movements reflect creativity rankings of the visualized motion trajectories as judged by an independent jury group. Additionally, we test the hypothesis that hand motion complexity might be a domain general indicator of an individual's creativity as measured by a range of simplified tasks requiring creative solutions that we adapted from typical psychometric creativity tests.

## Results

Participants in a *test group* were asked to generate motion patterns in virtual reality that they thought were as creative as possible. Crucially, each movement had to be repeated 10 times to ensure that creative patterns were planned and reproducible, rather than merely one-off chance events. In total, each participant in the test group was asked to generate 55 motion patterns that they considered to be as creative as possible. The motion patterns were recorded in a virtual reality set-up that consisted of a three dimensional Phantom manipulandum and a head-mounted display—see Materials and Methods for details. Participants were told that they had to repeat every movement 10 times by passing through a fixed sphere whose position in the workspace was determined randomly at the beginning of each trial. Otherwise, the movements could be chosen freely.

**Figure 1:**
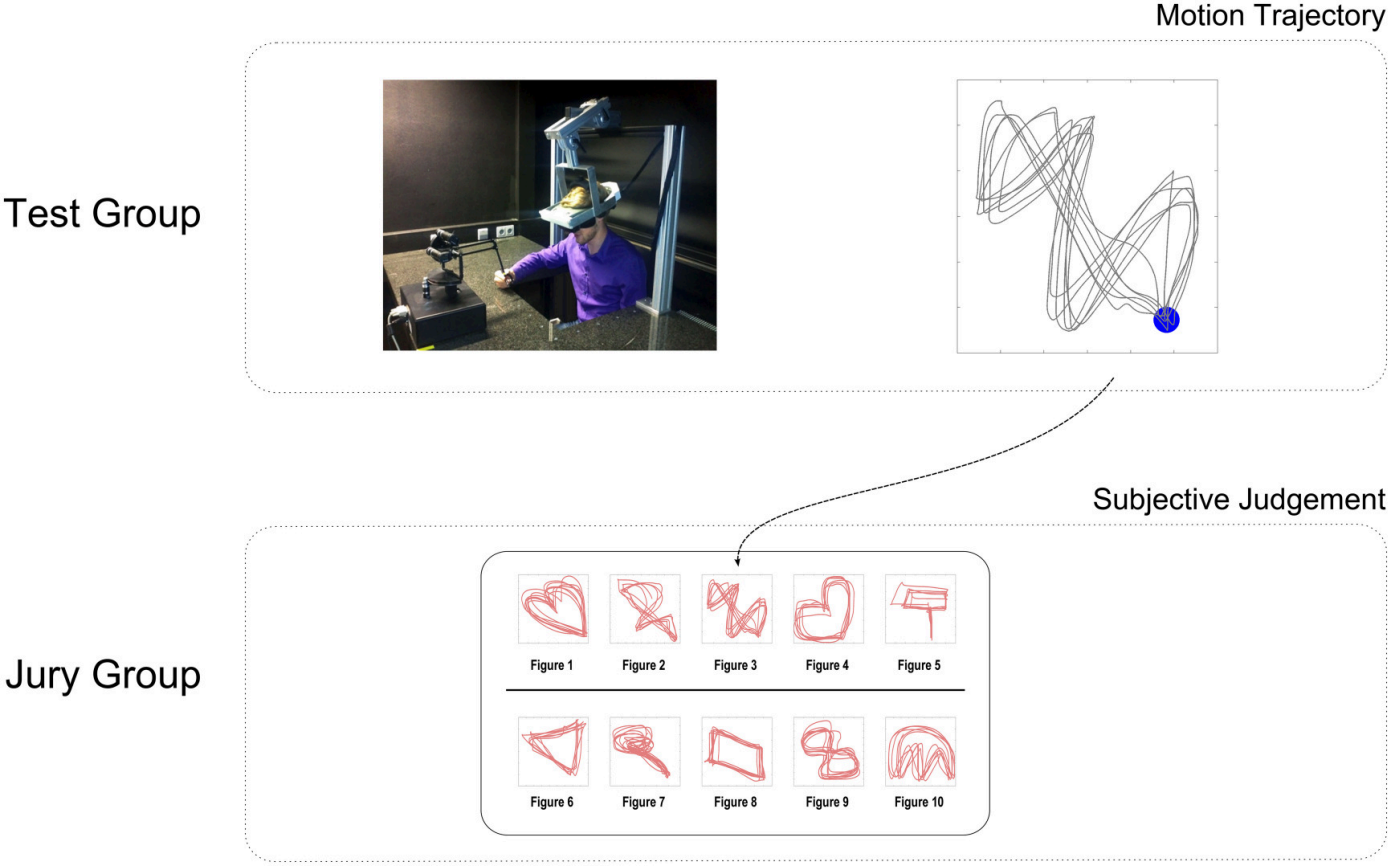
**Experimental design of a single trial**. Subjects in the test group were asked to generate motion patterns in virtual reality that they thought were as creative as possible. Each pattern consisted of 10 repeated trajectories. Subjects' movements were recorded with a manipulandum that constrained movements into the vertical plane within a workspace of 10 × 10 cm. Subjects saw their hand movement through a head-mounted display as a moving point in virtual reality. In total subjects were required to complete 55 trials, each trial consisting of a different motion pattern. A jury group was asked to rank visualizations of the test group's motion patterns according to their own subjective judgment of creativity.

After recording participants of the test group we invited another group of participants to act as a *jury group*. Each member of the jury group was asked to rank the visualized motion trajectories produced by the first group according to their subjective judgment of creativity. The ranking was established separately for each trial comparing the different motion patterns of the participants in the test group. As the test group consisted of 10 participants, the jury group could perform the creativity ranking by comparing no more than 10 items in each trial. An illustration of the experimental procedure can be found in Figure 1. The entire experiment was repeated twice to ensure reproducibility of the results.

### Motion complexity

As each motion pattern was repeated 10 times, we could use the corresponding 10 trajectories to estimate a probability distribution over trajectories. In particular, we used two different methods to estimate these probability distributions based on the recently proposed framework of Probabilistic Movement Primitives (Paraschos et al., 2013) and the well-established framework of Gaussian Processes (Rasmussen and Williams, 2006). In the framework of Probabilistic Movement Primitives, every single trajectory is transformed into a lower-dimensional representation. The distribution over repetitions, that is the pattern, is assumed to be a Gaussian distribution in the the space of lower-dimensional representations, which ultimately also implies a distribution in the space of trajectories—compare Equation (8) in the Materials and Methods. In the Gaussian Process framework, the distribution over trajectories is estimated directly based on a mean function and a covariance function between neighboring points of the trajectories. The length scale of the covariance function characterizes the trajectories' smoothness and complexity—compare Equation (3) in the Materials and Methods. The variance across repeated trajectories is assumed to be Gaussian. The advantage of having a probabilistic representation of the motion patterns is that it allows the application of information-theoretic complexity measures at the trajectory level without the need for a conversion into symbolic sequences. This way we can reduce motion complexity to the notion of model complexity used in Bayesian model comparison (Genewein and Braun, 2012, 2014).

Intuitively, complex models are flexible and can explain many data sets, whereas simple models can only explain few data sets. The fundamental trade-off of choosing the right level of model complexity can be seen most clearly if the model can be described by a number of parameters. A complex model has many possible parameter settings, which makes it probable that one parameter setting will be able to explain the data very well. However, there will be many parameter settings that will not be able to explain the data. In contrast, a simple model will only allow for few parameter settings and if one of these parameter settings fits well, there are fewer other parameter settings that cannot explain the data compared to the complex model. By computing the average data fit quality under all possible parameter settings the trade-off between goodness of fit and model complexity can be formalized. Mathematically, this is achieved by computing the marginal likelihood *p*(*D*|θ) that indicates the likelihood of some data *D* under model θ considering all possible parameter settings of this model. Importantly, under the two frameworks we are considering, the marginal likelihood can be written down analytically and has the following abstract form

(1) $$\log p(D|\theta )\propto -\underset{\text{goodness}-\text{of}-\text{fit}}{\underset{︸}{\frac{1}{2}x_{D}^{†}\Sigma^{-1}x_{D}}}-\underset{\text{model complexity}}{\underset{︸}{\frac{1}{2}\log|\Sigma |}}$$

**Figure 2:**
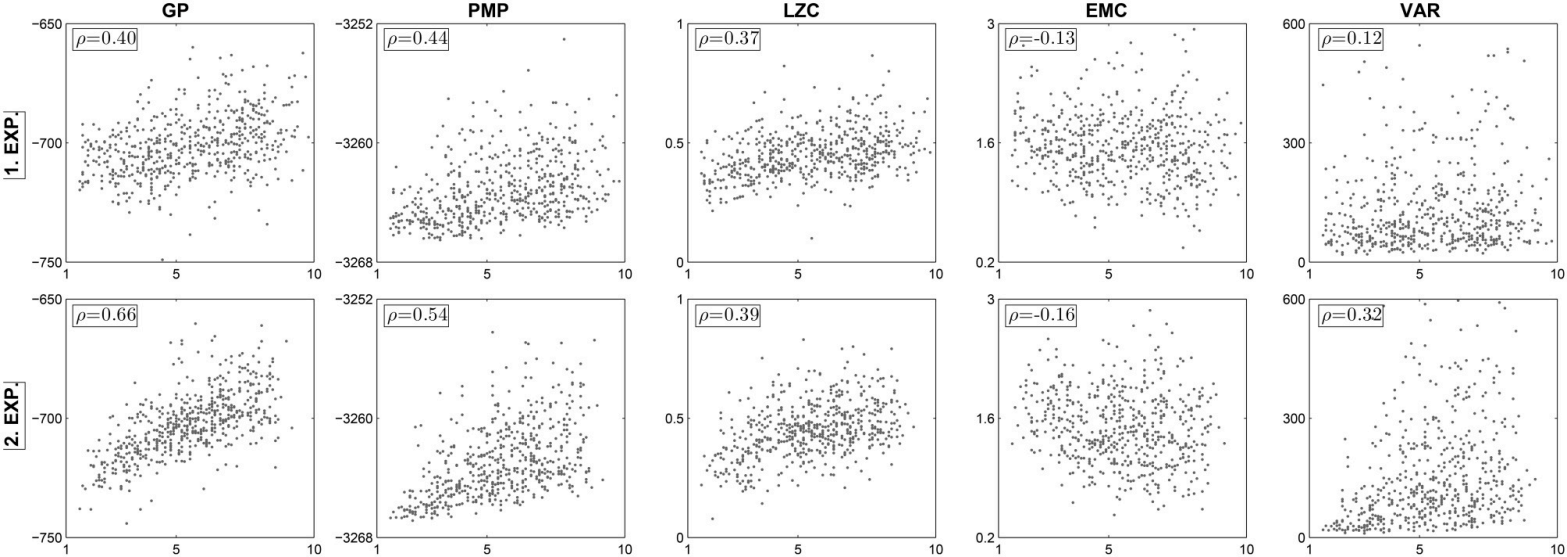
**Scatter plots of subjective creativity rankings and motion complexity measures**. In all 10 panels the abscissa shows average subjective creativity ranking scores attributed to motion trajectories generated by subjects of the test group. The five different columns show complexity values for the Gaussian Process model (GP), the Probabilistic Movement Primitive framework (PMP), the Lempel-Ziv complexity (LZC), the effective measure complexity (EMC), and the trajectory variance (VAR), respectively. The two rows present the results of the two experimental repetitions. Five hundred fifty data points are shown in each panel plot, as we collected 55 patterns from each of the 10 subjects in each experiment.

The complexity term corresponds essentially to the entropy of a Gaussian distribution. Intuitively, the entropy measures the average amount of informational surprise that each pattern contains. Here this informational surprise can therefore be applied to capture the informational complexity of motion patterns. The informational complexity scores achieved by subjects under the two frameworks can be seen in the first two columns of Figure 2.

In line with our previous study, we also computed two other measures of motion complexity, namely Lempel-Ziv complexity and effective measure complexity—see Materials and Methods for details. These complexity measures are typically applied to sequences of symbols. We therefore converted subjects' motion trajectories into symbol sequences by parcellating the 10 × 10 cm workspace into evenly spaced 1 *cm*^2^ grid cells. The motion trajectory could then be converted into a sequence of up, down, left, and right cell transitions. By sampling multiple trajectories from each motion pattern distribution fitted by the probabilistic movement primitives, we obtained symbol sequences of sufficient length to compute both Lempel-Ziv complexity and effective measure complexity for each pattern. Intuitively, the (normalized) Lempel-Ziv complexity quantifies the irregularity in a symbol sequence based on the number of unique subsequences, while effective measure complexity unravels the structural complexity of a movement that is not too random, but also not too stereotypical. The symbol-sequence complexity scores achieved by subjects can be seen in the third and fourth column of Figure 2.

### Subjective creativity judgment

To obtain independent creativity measurements, we invited a jury group to assess visual displays of the hand motion patterns produced by the test group. The first 10 jurors judged patterns produced by the first 10 participants in the test group, while the last 10 jurors were assigned to judge the patterns from the last 10 subjects in the test group. Accordingly, each juror had to compare 10 patterns in each trial from 10 different subjects and rank them according to their own subjective concept of creativity—compare Figure 1 for an example. Jurors had to complete 55 trials in total. Then we could compute the average rank for each trial by averaging the rankings scores of all jurors. Finally, we could average the rankings of all jurors across all trials to obtain an overall rank for each test subject.

We compared the average creativity rankings with the four motion complexity measures introduced in the last paragraph. Figure 2 shows the trial-by-trial scatter plot of these measures in both experiments. In all plots, the abscissa indicates the average rank of a test subjects' pattern in a particular trial, while the ordinate shows the corresponding motion complexity measure of the pattern. With the exception of the effective measure complexity, we found reproducible correlations between all the informational complexity measures and the subjective creativity judgments. The motion complexity in the Gaussian Process model had a Spearman's rank correlation with the creativity rankings of ρ = 0.40 (*p* < 10^−10^) and ρ = 0.66 (*p* < 10^−10^) across the two experiments. The motion complexity in the Probabilistic Movement Primitive framework showed a Spearman's rank correlation with ranked creativity of ρ = 0.44 (*p* < 10^−10^) and ρ = 0.54 (*p* < 10^−10^) across the two experiments. When representing trajectories as symbol sequences, we also found a moderate correlation between the Lempel-Ziv complexity of motion patterns and subjective creativity judgments in both experiments (ρ = 0.37 with *p* < 10^−10^ and ρ = 0.39 with *p* < 10^−10^). The effective measure complexity only showed a negligible correlation with the creativity rankings—compare Figure 2.

**Figure 3:**
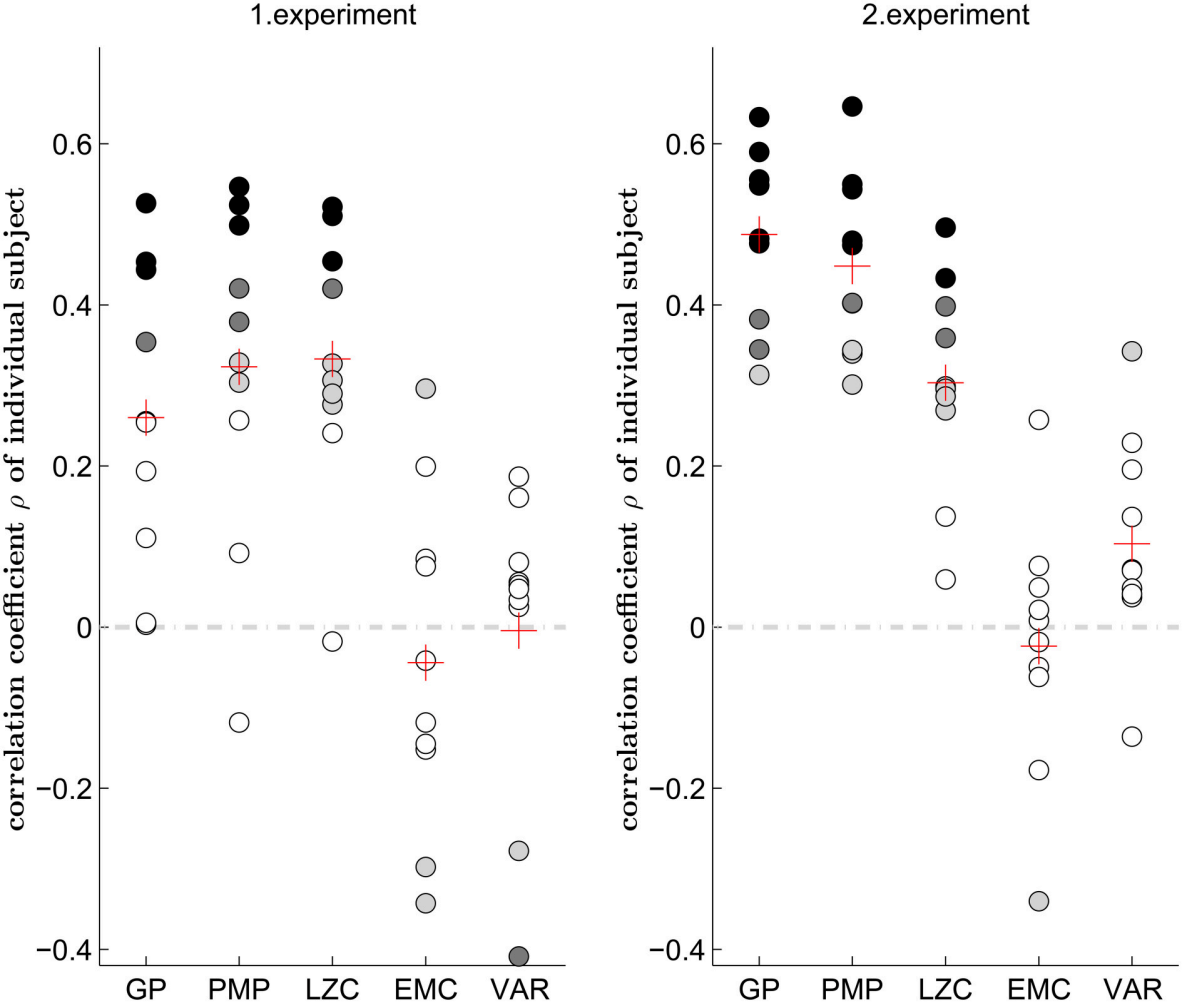
**Correlation between motion complexity measures and subjective creativity judgment for each single subject**. Five measures are compared, they are complexity under the Gaussian Process model (GP), the complexity under the framework of Probabilistic Movement Primitives (PMP), Lempel-Ziv complexity (LZC), effective measure complexity (EMC), and variance (VAR). Each dot represents the Spearman's rank correlation coefficient of one subject. A filled dot indicates a significant correlation (black: *p* < 0.001, dark gray: *p* < 0.01 and light gray: *p* < 0.05) and a white dot indicates that the correlation is not significant (*p* > 0.05). The red cross indicates the average correlation coefficient over all subjects.

Importantly, the correlation between the entropic motion complexities and subjective creativity rankings were also significant at the single subject level, that is when assessing correlations between motion complexity and subjective creativity judgments of the 55 trials generated by each single subject—compare Figure 3. In the Gaussian process model, we found significant correlations between entropic complexity and subjective creativity judgments in 14 out of 20 subjects. Correlating entropic complexity and subjective creativity judgments in the framework of Probabilistic Movement Primitives resulted in significant correlations in 17 out of 20 subjects. When transforming trajectories into symbol sequences, we found a significant correlation between Lempel-Ziv complexity and subjective creativity judgments in 16 out of 20 subjects. In contrast, we found no consistent correlation for effective measure complexity.

**Figure 4:**
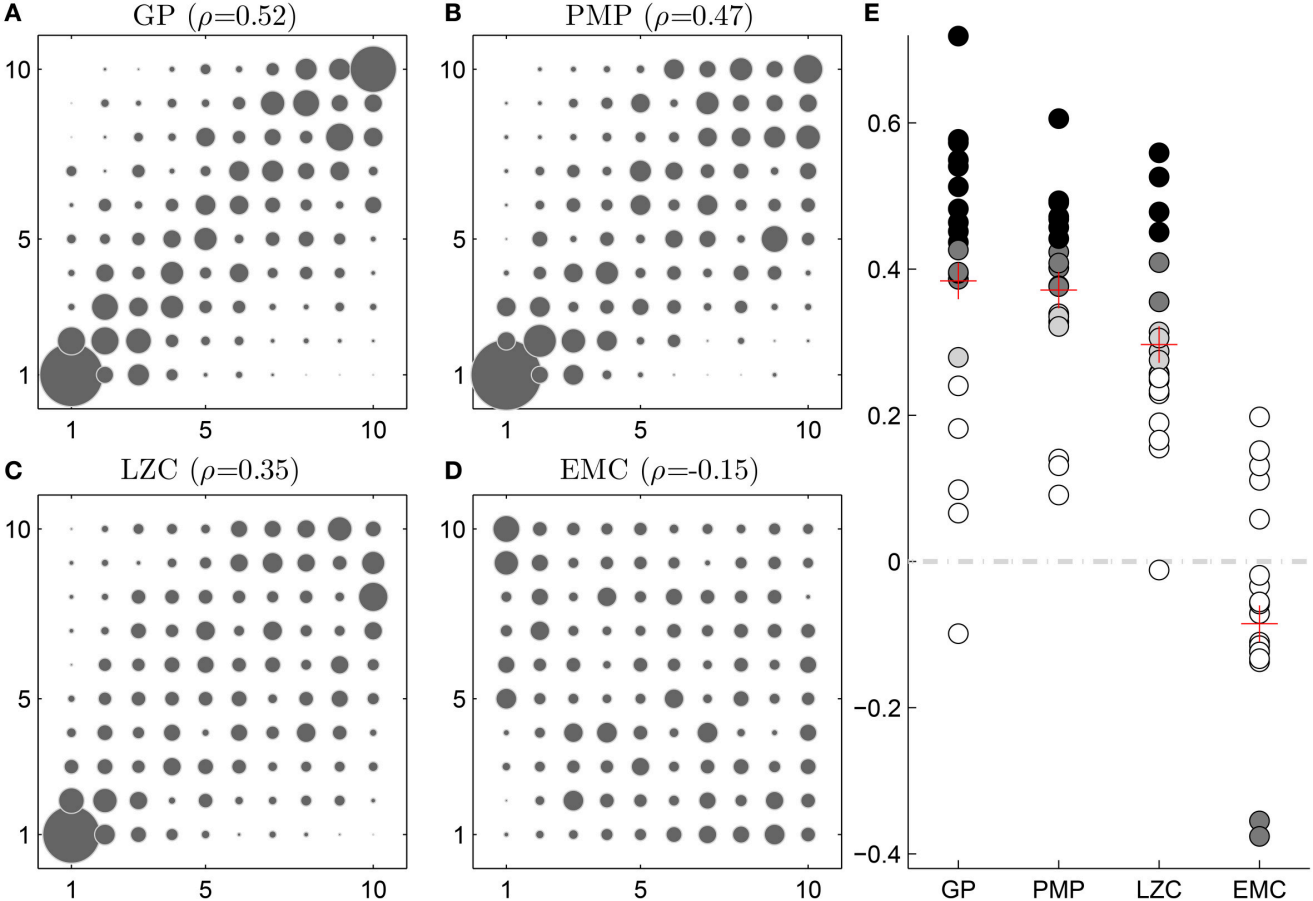
**Ranking results**. **(A–D)** 2d histograms of rank pairs consisting of subjective creativity rankings and different motion complexity rankings. With exception of effective measure complexity (EMC), the other informational complexity rankings have most coincidence with the creativity rankings on the diagonals. **(E)** Subject-wise correlation between creativity rankings and complexity rankings. The rank-rank correlation is significant in the case of complexity under the Gaussian Process model (GP) for 15 out of 20 subjects, in the case of the complexity under the framework of Probabilistic Movement Primitives (PMP) for 17 out of 20 subjects, in the case of Lempel-Ziv complexity (LZC) for 10 out of 20 subjects and in the case of EMC for 3 out of 20 subjects.

Furthermore, the same patterns of correlation persist when replacing the absolute complexity values with complexity ranks. To this end we rank ordered according to complexity the 10 relevant motion patterns in each trial that were also shown to jurors. Moreover, we replaced the average subjective creativity rank by a full rank 1, 2, …, 10. This way we can ensure that the correlations we found for absolute complexities also hold for ranks. The rank-rank correlations between complexity and creativity can be seen in Figure 4.

As each motion pattern had to be repeated 10 times, due to imperfect memory more complex trajectories might have been naturally accompanied by higher variability. This way our data set might show an intrinsic correlation between variability and complexity. The different randomness measures that correlate with subjective creativity might therefore only be a side effect of the increased variance of complex patterns. To control for this possibility, we computed the variance across the 10 original trajectory repetitions. The fifth column in Figure 2 shows the trial-wise correlation between the trajectory variance and the subjective creativity judgment. We only found a weak correlation in both experiments. Figure 3 also shows the correlation between the trajectory variance and the subjective creativity judgment at the single subject level. Only 3 out of 20 subjects revealed significant correlation, but with inconsistent signs. This result suggests that the correlations between entropic complexity measures and creativity rankings do not simply reflect variance across trajectories.

### Additional creativity assessments

Participants in the *test group* additionally accomplished five creativity tasks adapted from the psychological literature including the *Creativity Achievement Questionnaire* (CAQ), the *Alternative Uses Task* (AUT), the *Cloud Imagination Task* (CIT), the *Picture Completion Task* (PCT), and the *Remote Associates Test* (RAT)—see Materials and Methods for details. These tasks were meant to assess both subjects' creative achievement (CAQ) and creative potential (AUT, CIT, PCT, RAT). In particular, the AUT, the CIT and the PCT were meant to measure subjects' divergent thinking, while the RAT is thought to reveal individual ability of remote association. The results of these creativity tests are shown in Supplementary Figure 1.

We investigated the correlation between the different motion complexity measures and the five creativity assessments of each individual. The results of this analysis are shown in Table 1. We compared the average achieved motion complexities over 55 trials of each subject with the achieved scores in the creativity tasks we used in our study. As the process of generating hand movement might involve spatial visualization and hand motion ability, we also used four sub-scales in the CAQ as additional metrics, namely art, music, dance, and theater/film. Although architectural design also requires visual and drawing skills, only two subjects in the test population claimed they have received training in this domain, therefore, we excluded this sub-scales from our analysis. For the divergent thinking tests we used three different metrics, namely fluency, mean originality and percentage score—see Materials and Methods for details. However, we found no consistent correlation between any pair of measures, that is no correlation that could be consistently reproduced over two runs of the experiment. Finally, we investigated the correlation between the overall subjective creativity rank of a subject and the achieved scores in the pen-and-paper creativity tasks. The results are shown in Table 1. Again we found no significant correlation between subjective creativity judgments and the achieved scores in the creativity tests.

**Table 1:** **Spearman's rank correlation coefficient between motion complexity measures and creativity assessments of an individual**.

		**GP**		**PMP**		**LZC**		**EMC**		**Ranking**	
		**ρ**	***p*-value**	**ρ**	***p*-value**	**ρ**	***p*-value**	**ρ**	***p*-value**	**ρ**	***p*-value**
CAQ	Total	0.50	0.03	0.26	0.27	0.16	0.50	−0.12	0.63	0.45	0.05
	Art	0.19	0.43	−0.16	0.50	−0.06	0.81	0.09	0.70	0.32	0.17
	Music	0.26	0.27	0.10	0.68	−0.08	0.73	0.07	0.77	0.28	0.24
	Dance	−0.19	0.42	−0.24	0.31	−0.30	0.20	0.32	0.16	−0.08	0.75
	Theater	0.09	0.69	−0.10	0.68	−0.27	0.26	0.23	0.33	−0.13	0.57
AUT	Fluency	−0.23	0.33	−0.43	0.06	−0.33	0.15	0.23	0.32	−0.11	0.65
	m. Orig.	0.12	0.62	−0.06	0.79	−0.05	0.83	0.09	0.70	0.23	0.33
	Percent.	0.04	0.86	−0.11	0.63	−0.19	0.43	0.16	0.50	−0.13	0.60
CIT	Fluency	−0.04	0.86	0.12	0.60	0.26	0.27	−0.37	0.11	−0.24	0.31
	m. Orig.	0.02	0.95	0.24	0.31	0.24	0.31	−0.38	0.10	−0.08	0.75
	Percent.	0.01	0.96	0.30	0.20	0.37	0.10	−0.50	0.03	−0.00	0.99
PCT	Fluency	−0.09	0.71	−0.24	0.30	−0.05	0.84	0.01	0.95	−0.12	0.62
	m. Orig.	0.07	0.76	0.36	0.12	0.26	0.26	−0.18	0.44	−0.05	0.85
	Percent.	0.04	0.87	0.33	0.15	0.29	0.21	−0.18	0.44	−0.05	0.84
RAT		0.39	0.09	0.27	0.25	0.25	0.28	−0.01	0.95	0.35	0.13

We found no consistently significant correlation. The first four columns show correlations between performance in the creativity tasks and the complexity measures in a Gaussian Process Model (GP), within the framework of Probabilistic Movement Primitives (PMP), Lempel-Ziv complexity (LZC), and effective measure complexity (EMC). The last column shows the correlation between subjective creativity ranking of the visualized motion trajectories and the results of the creativity tasks. The p-values are not Bonferroni-Holmes corrected. After correction there are no significant correlations.

## Discussion

In this experimental study we have tested the hypothesis that the motion complexity individuals generate in their movements might be correlated to the degree of creativity assigned by a human observer to the visualized motion trajectories. In addition to our previous complexity analysis that required translating movements into symbol sequences (Peng et al., 2014), in this study we adopted state-of-the-art machine learning approaches—Gaussian Processes (Rasmussen and Williams, 2006) and Probabilistic Movement Primitives (Paraschos et al., 2013)—to represent movement patterns directly as probability distributions over trajectories. This allowed us to assess motion complexity in terms of Bayesian model complexity, which corresponds to informational surprise (Genewein and Braun, 2014). The link between creativity and information surprise has been previously proposed by Schmidhuber (2010, 2012). In our data we found a significant and reproducible correlation between the information-theoretic complexity measures of subjects' motion trajectories and the subjective creativity judgment of independent jurors. We did not find any consistent correlation between motion complexity measures and creativity measures that we obtained in a set of adapted divergent thinking and remote associate tasks as well as creative achievements scores from a questionnaire.

In a previous study (Peng et al., 2014) we have shown the applicability of symbol-based randomness and complexity measures to human hand motion. In order to apply these measures, we converted motion trajectories into symbolic sequences *s*_1_*s*_2_*s*_3_…*s*_*n*_, with *s*_*i*_ ∈ {*l, r, u, d*} corresponding to “left,” “right,” “up,” and “down” movements. Due to the limited number of symbolic cell transitions in short movements, we were previously unable to estimate different complexity measures of single motion patterns, and we could only estimate complexity measures of concatenated movements. We have overcome this limitation in the present study by representing movement patterns as probability distributions over trajectories, which we exploited in two ways. First, we determined motion complexity of movement patterns directly by applying the concept of Bayesian model complexity to the probabilistic trajectory representation. Second, we applied the previous symbol-based complexity measures to single patterns by generating an arbitrary amount of samples from the trajectory distributions.

**Figure 5:**
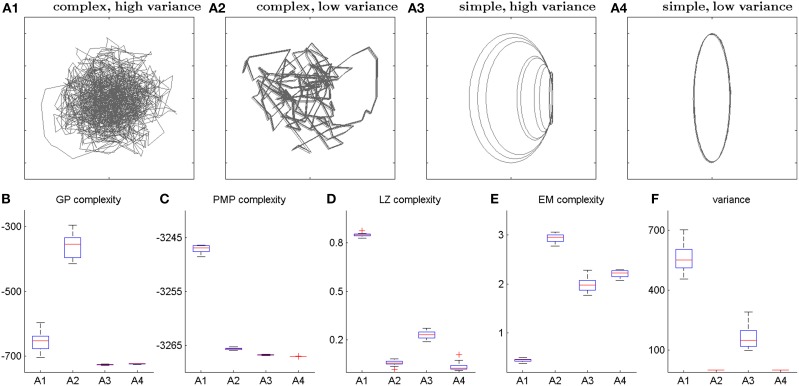
**Artificial example trajectories**. **(A1–A4)** Panel **(A1)** shows 10 trajectory realizations of a random pattern with large variability. Panel **(A2)** shows 10 trajectory realizations of a complex pattern with low variance. Panel **(A3)** shows 10 trajectory realizations of a simple elliptic pattern with large variability between the trajectories. Panel **(A4)** shows a simple elliptic pattern with little variance. We have generated 20 patterns of each type. **(B–F)** Box plots of motion complexity measures of artificial trajectories. Panels **(B–E)** show informational complexity of the artificial trajectories in the Gaussian Process model **(B)**, the Probabilistic Movement Primitive framework **(C)**, the Lempel-Ziv complexity **(D)**, and the effective measure complexity **(E)**. Panel **(F)** shows the trajectory variances.

A main concern regarding the proposed complexity measures is that the measured information-theoretic surprise might simply reflect trial-by-trial variability. In our experimental setup this issue is particularly crucial, as subjects were asked to repeat each movement 10 times and trial-by-trial variability can be expected to be increased for complex movements due to imperfect memory. Importantly, we found in our data that this is not the case, and that the complexity measures do not simply reflect trial-by-trial variability—compare Figure 2. This can also be illustrated in artificially generated trajectories with different levels of complexity and variability—compare Figure 5. Both the Gaussian Process and the Probabilistic Movement Primitives complexity measures do not simply reflect variability, as both measures are higher for complex-low variance trajectories (Figure 5A2) than for simple-high variance trajectories (Figure 5A3). However, for completely random movements the Gaussian Process complexity is low, because observations can be explained by a smooth trajectory with high noise. In contrast, the movement primitive complexity is high, because a broad distribution in feature space is required to represent this trajectory distribution. Notably, this extreme case of complete randomness is irrelevant in our experiment and both frameworks make similar predictions for repeatable trajectories that are not completely random. In contrast to these probabilistic measures, the Lempel-Ziv complexity does follow the variance pattern in these artificial data sets. The reason is that Lempel-Ziv complexity does not distinguish between irregularity in an individual trajectory and variability across trajectories. In our previous study this did not constitute a problem, because there were no repeated trajectories. In the present study, however, this issue has to be kept in mind when interpreting the Lempel-Ziv measure, as it is most sensitive to the variability, even though the data analysis shows that it gives complexity information beyond variability—compare Figure 2. Note that, while the effective measure complexity classifies the artificial data well, it did not yield any significant correlations with subjective creativity rankings.

A number of previous studies have established a link between physical movement and human creativity. One study has shown that mood and creativity can be improved by physical exercise independently of each other (Steinberg et al., 1997). Another study recently showed that fluid arm movements can enhance creativity in alternative use tasks, cognitive flexibility, and remote association (Slepian and Ambady, 2012), suggesting that fluid movement might improve fluid thinking, whereas abrupt movements do not. Similarly, another recent study revealed that walking indoors or outdoors boosted creative inspiration. Creativity levels were consistently and significantly higher for those walking compared to those sitting (Oppezzo and Schwartz, 2014). While these studies show that bodily movement can actually influence cognitive processing and enhance human creativity, none of the previous studies have shown a relation between the complexity of the movements and subjects' creative potential.

In the literature (Mumford, 2003; Hennessey and Amabile, 2010), the definition of creativity is still a topic of ongoing debate, although most researchers agree that “*creativity involves the development of a novel product, idea or problem solution that is of value to the individual and/or the larger social group”* (Hennessey and Amabile, 2010). Creativity in the sense of “*novelty and appropriateness”* can be displayed both in daily problem solving (“Little c”: everyday creativity) and in lifetime achievements that have a major impact on others (“Big C”: eminent creativity). Boden (2004) introduces a similar distinction of p-creativity (personal) and h-creativity (historical), respectively. Recently, the “Two C” distinction has been extended to the “Four C Model” by Kaufman and Beghetto (2009)—besides “Little c” and “Big C,” “mini c” indicates the creativity inherent in the learning process and “Pro C” represents the professional-level expertise in any creative area.

The creativity tasks that we adapted from established psychometric creativity tests were selected on the basis that the tasks should be easy to implement and straightforward to evaluate in the absence of trained experts. However, this simplification might have caused some drawbacks in our experimental design. First, using only one single item for the Alternative Use Task is underpowered compared to the whole battery of Guilford's Alternative Uses Task (Guilford, 1967). Second, the reliability and validation of the Cloud Imagination Task and the Picture Completion Task have not been systematically verified. Third, the German version of the Remote Association Task was not independently validated. These experimental flaws may be one of several possible reasons for why we did not find any significant correlation between motion complexity and the results of our adapted creativity tasks.

Furthermore, even the evaluation of the established psychometric creativity tests are still under debate, especially some of the scoring systems (Mumford et al., 2008). For example, instead of determining originality and fluency scores in the alternative use task, Runco and Mraz have proposed to evaluate responses based on ratings of subjects' replies through additional judges (Runco and Mraz, 1992). Silvia and colleagues have proposed the “Top 2” scoring method in which participants in divergent thinking tests have to chose two of their own responses that they consider as most creative, judges then evaluate the responses on a 5-point scale (Silvia et al., 2008). Similarly, the RAT was criticized by Worthen and Clark with the main argument that RAT measures sensitivity to language rather than creative potential (Worthen and Clark, 1971). They proposed accordingly an improved version called Functionally Remote Associates Test (FRAT) that keeps the same solutions as used in the original RAT task, however, rebuilds the stimulus words based on functional associations rather than verbal associations. Although the validity and reliability of existing methodologies to assess individual creativity is a topic of debate, they are still widely applied (Plucker and Runco, 1998; Plucker, 1999). According to Baer (1998, 2008), a fundamental problem with divergent thinking tests and their fragile results might be that creativity could be highly domain-specific. Consequently, there may not exist any single monolithic cognitive process underlying creativity that could be measured reliably by divergent thinking tests or any other one-dimensional test metric (Dietrich, 2007). This could also be a possible explanation for why we did not find any correlation between motion complexity and several creativity tasks in our experiment.

A recent study by Lee and colleagues (Lee et al., 2014) showed that the scores on the RAT were positively and significantly correlated with scores on working memory and intelligence tasks, and were not significantly correlated with scores on indicators of divergent thinking test. Therefore, the RAT may be more likely to be an assessment of intelligence rather than creativity. Also in our motion task we cannot exclude a correlation between working memory, intelligence and motion complexity. In fact, the correlation between creativity and intelligence has been debated for a long time across a wide range of domains (Silvia, 2008; Jauk et al., 2013). What our data clearly shows, however, is that motion complexity correlates with subjective creativity rankings of the visualized motion patterns. This assessment by averaging creativity rankings differs from usual consensual assessment techniques in that we forced jurors to rank order specimens rather than assigning absolute levels of creativity. The reason we introduced this ranking procedure was to avoid a floor effect, where absolute creativity levels of most motion patterns might be judged below a critical creativity threshold.

Over the last decade, creativity has become an exciting research topic across a number of disciplines ranging from psychology and cognitive science, to neuroscience and artificial intelligence. Modern neuroscientists have attempted to reveal the neural processes in the brain underlying creative thought by means of advanced neuroimaging technologies like functional magnetic resonance imaging (fMRI). Vandervert and colleagues have argued, for example, that the cerebellum may play a crucial role in creative thinking as it increases the rapidity and efficiency of memory routines (Vandervert et al., 2007). As the cerebellum is crucial for motor control, this study also suggests that there might be a link between motion complexity and creativity. Recent reviews of neuroimaging studies of creativity can be found for example in Dietrich and Kanso (2010) and Arden et al. (2010).

While some researchers are trying to understand how creative thinking is generated in human brains, other researchers are attempting to implement computational creativity in artificial systems, including linguistic processing (Binsted et al., 1997), composition of music (Gibson and Byrne, 1991), and artistic creation (Romero and Machado, 2008). From a computational creativity perspective, Boden proposed three types of creativity, namely combinational, exploratory and transformational creativity (Boden, 1998). Combinational creativity involves novel combinations of familiar ideas, exploratory creativity is generated by exploring structured conceptual spaces, and transformational creativity is revealed by transforming some dimension of an existing space to create novel structures. The existing artificial systems with apparent artificial creativity (Gibson and Byrne, 1991; Binsted et al., 1997; Romero and Machado, 2008) based on genetic algorithms or boosted by a bootstrapping process can also be seen as exhibiting exploratory and combinational creativity under Boden's definition. Schmidhuber's description of creativity as finding novel patterns and regularities that allow for improved data compression is maybe the most abstract characterization that can be thought to subsume these different types of creativity. Our study provides evidence for this link between creativity and informational complexity in the domain of hand motions.

## Materials and methods

### Participants and apparatus

Forty participants took part in the study. All participants were naïve and gave informed consent before starting the experiment. The study was approved by the ethics committee of the Max Planck Society. Motion trajectories were recorded in three dimensions by means of a virtual reality setup consisting of a Sensable® Phantom® Premium 1.5 High Force manipulandum and an NVIS® nVisor ST50 head-mounted display (HMD) for creating stereoscopic 3D virtual reality—see Genewein and Braun (2012) for details.

### General experimental procedure

Participants were assigned into two groups, which we refer to in the following as the *test group* and the *jury group*. Each group consisted of twenty subjects. The first twenty participants were assigned to the *test group* and they were asked to accomplish several creativity tests and complete a drawing task in virtual reality. The last twenty participants were assigned to the *jury group* and were asked to judge the drawings produced by the first group. Figure 1 shows the experimental procedure of both groups.

#### Test group

Experimental procedures in this group comprised two sessions. In the first session, subjects were asked to complete five different creativity tasks by writing their answers on paper.

- *Creativity Achievement Questionnaire* (CAQ). The CAQ is a self-report measure of creative achievement developed by Carson et al. (2005). Participants are asked to rank their own creative achievements from 0 to 7 in ten different domains: visual arts, music, dance, architectural design, creative writing, humor, inventions, scientific inquiry, theater/film, and culinary arts. The test sheet used in our study can be found at the end of Carson's paper (Carson et al., 2005).
- *Guilford's Alternative Uses Task* (AUT) for divergent thinking (Guilford, 1967). Subjects were asked to write a list of as many uses for a “newspaper” as possible in 1 min.
- *Cloud Imagination Task* (CIT). In this task we showed subjects a single picture of a natural cloud and asked them to write down as many interpretations of the cloud as they could think of within 1 min.
- *Picture Completion Task* (PCT). In this task we showed subjects two non-aligned circles and asked them to conjure up images where the circles would form a natural part of the image. Rather than drawing these images as proposed by Torrance (1974), we asked subjects to write down short descriptions of their imaginations. Again, this test had to be completed in 1 min.
- *Remote Associates Test* (RAT; Mednick and Mednick, 1967). In each trial, three seemingly unrelated words were shown on a computer screen, and subjects had 25 s to find a single word that related to each of these three words. In total, there were 15 trials per subject with varying difficulty. For the English version of the test we selected 15 questions from the online collection of RAT questions at Berkeley University^1^. For the German version of the test we used the same 15 solutions and modified the three stimulus words accordingly.

The first session started with the CAQ, where subjects filled out the questionnaire in paper form. The four following tests were displayed on a computer screen and subjects gave their answers in written form on a separate piece of paper. At the beginning of each test, the experimenter read the instructions on the screen aloud and provided further information to the subjects if required. Only when subjects indicated that they understood the test requirements, they were allowed to continue by clicking the “next” button to start the test. The button click triggered a timer. When reaching the time limit, a slide indicating the end of the test was shown and a beep occurred to inform subjects that the test was over. Subjects were not allowed to write anything more on the answer sheet after the beep. After 5 s the next test instructions were displayed.

In the second session, subjects operated a 3*D* manipulandum in a virtual reality environment where they controlled a cursor (blue, radius 4 mm) representing their hand position. In each trial, their task was to generate 10 repetitions of a self-chosen trajectory in the vertical plane in a 10 × 10 cm workspace that was displayed in 3*D*. Subjects could not move outside the grid as they were constrained by a spring force to stay within the vertical plane and within the boundaries of the grid. The spring constant was set to 8 N/cm. To initiate the trial subjects had to move to a start sphere (blue, radius 6 mm), which was randomly placed in the grid at beginning of the trial. In each trial, subjects were requested to draw a closed-form pattern and to repeat this pattern 10 times. The pattern had to include the start sphere and the trial was completed when passing through the start sphere for the tenth time. Importantly, subjects only ever saw their current hand position displayed and had to remember the pattern over the course of the repetitions. In total, each subject completed 55 trials. They were explicitly instructed to be as creative as possible.

#### Jury group

The first 10 jurors were asked to judge the motion trajectories produced by the first 10 participants in the test group, while the last 10 jurors judged motion trajectories from the last 10 participants in the test group. The jury group were informed that a previous group of subjects performed in a free motion generating task with the instruction to be as creative as possible. They were told that now their job was to rank these visualized motion trajectories collected from the previous experiment according to their subjective judgment of creativity. At any one time, they compared 10 motion patterns that were produced by the 10 previous subjects in the same trial. The 10 motion patterns were displayed on a computer screen as illustrated in Figure 1. On the screen the 10 motion patterns were randomly arranged to rule out spatial preference effects. The motion patterns were shown in consecutive order of the trial number. Subjects of the jury group were asked to write down the rank of the motion patterns from most creative (rank 10) to least creative (rank 1). There was no time limit for the jury group.

### Data analysis

#### Metrics for divergent thinking tests

Three metrics were used to evaluate the responses of the divergent thinking tests, namely *fluency, percentage*, and *mean originality*. Fluency is simply defined as the total number of meaningful and interpretable responses produced by a subject. The originality score is conventionally defined as the number of responses provided by <20% of the samples. However, this metric is not independent from fluency. Accordingly, the percentage scoring method, which is computed as originality scores divided by fluency scores, has been suggested as a more appropriate scoring strategy for divergent thinking tests rather than the conventional originality scores (Plucker, 2011). Mean originality is another measure, which has demonstrated good discriminative validity (Zenasni and Lubart, 2009). There are three steps to calculate the mean originality scores: (1) compute the relative frequency of each response provided by subjects; (2) compute one minus the frequency, so that the rarer response yields a higher score; (3) sum up all scores of responses in one subject and divide it by the number of responses generated by this subject. This average score is defined as the mean originality of a single subject.

#### Gaussian process model

Gaussian Processes are a well-established technique in machine learning to represent probability distributions over functions. In our case the functions are movement trajectories indexed by time. Accordingly, a Gaussian process over time can be specified as

(2) f(t)~GP(m(t),k(t,t′))

where *m*(*t*) is the mean function, and *k*(*t, t*′) indicates the covariance function. In our study, we use a zero-mean prior *m*(*t*) = 0 and assume a squared exponential covariance function

(3) $$k(t,t\prime )=\sigma_{f}^{2}\cdot \exp\left(-\frac{|t-t\prime {|}^{2}}{\lambda^{2}}\right)$$

where σ_*f*_ is the signal variance and λ is the characteristic length scale. For each trajectory *T* = 150 equidistant points are chosen in time which defines a vector *t* = [1, 2, …, *T*]. The two movement dimensions of a motion trajectory are defined by the corresponding vectors *x* = [*x*_1_, …, *x*_*T*_] and *y* = [*y*_1_, …, *y*_*T*_]. As movements were two-dimensional, we treated each dimension as a separate Gaussian Process with length scales λ_*x*_ and λ_*y*_. For a given length scale λ, the covariance function at the time points *t* can be abbreviated as a matrix *K*_λ_ = *k*_λ_(*t, t*). For any particular pattern we concatenated the 10 observed trajectories into one single trajectory. Observed trajectories are assumed to be noisy samples of the truly intended trajectory, where for example in the y-dimension *y*_*i*_ = *f*(*t*_*i*_) + ϵ_*i*_ with $\epsilon_{i}\sim N\left(0,\sigma_{n}^{2}\right)$. As shown in Equation (2.30) in Rasmussen and Williams (2006) the marginal likelihood for a given set of observation can be expressed as

(4) $$\log P(y|t,\lambda )=-\underset{\text{goodness}-\text{of}-\text{fit}}{\underset{︸}{\frac{1}{2}y^{†}{\left(K_{\lambda }+\sigma_{n}^{2}I_{N}\right)}^{-1}y}}-\underset{\text{model complexity}}{\underset{︸}{\frac{1}{2}\log\det\left(\frac{K_{\lambda }+\sigma_{n}^{2}I_{N}}{2\pi }\right)}}$$

where † denotes the transpose operation. Similarly, the complexity of the other movement dimension *x* is computed. For the total complexity the two dimensions are added, where the overall marginal likelihood is given by log*P*(*x, y*|*t*, λ_*x*_, λ_*y*_) = log*P*(*x*|*t*, λ_*x*_) + log*P*(*y*|*t*, λ_*y*_). The data-independent model complexity is entirely determined by the length scale λ. For each movement pattern λ was fitted so as to maximize the marginal likelihood. Based on this maximum likelihood λ we could then compute the trajectory complexity.

#### Probabilistic movement primitives

Movement primitives are a well-established approach for representing modular building blocks of movements with applications both in computational neuroscience and robotics (Schaal et al., 2003). Recently, Paraschos and colleagues proposed a probabilistic formulation of movement primitives where a primitive represents a distribution over trajectories (Paraschos et al., 2013). Here we adopted this approach to represent human drawing patterns.

A single movement is denoted by a trajectory τ = {*q_t_*} _*t*=0⋯*T*_ where *q*_*t*_ = [*x*_*t*_; *y*_*t*_] defines the end effector position at time *t*, and *x*_*t*_ and *y*_*t*_ refer to the two dimensions of the vertical plane. Given an *n*-dimensional time-dependent feature vector Φ_*t*_, it is assumed that the trajectory can be represented compactly by a linear combination of features with weight vector ω such that

(5) $$\begin{array}{l} q_{t}=\left[\begin{array}{c} x_{t}\\ y_{t} \end{array}\right]=\left[\begin{array}{cc} \Phi_{t}^{†} & 0_{1\times n}\\ 0_{1\times n} & \Phi_{t}^{†} \end{array}\right]\;\left[\begin{array}{c} \omega_{x}\\ \omega_{y} \end{array}\right]+\epsilon_{q}=\Psi_{t}\omega +\epsilon_{q},\\ \;p(q_{t}|\omega )=N(q_{t}|\Psi_{t}\omega ,\Sigma_{q}) \end{array}$$

where ϵ_*q*_ ~ N(0, Σ_*q*_) is zero-mean Gaussian noise. The variable Ψ represents a block-diagonal matrix composed of the feature vectors. Moreover, we assumed the feature matrix Φ_*t*_ to be composed of Gaussian basis functions given by

(6) $$b_{i}(z_{t})=e^{-\frac{{\left(z_{t}-c_{i}\right)}^{2}}{2h^{2}}},\;\phi_{i}(z_{t})=\frac{b_{i}(z_{t})}{\sum_{j=1}^{n}b_{j}(z_{t})}$$

where *h* defines the width and *c*_*i*_ the center for the *i*th basis function. In our implementation we set *n* = 20, that is ω ∈ ℝ^40^. The phase variable *z*_*t*_ is introduced to transform the temporal variable *t* into phase space. In general, *z*(*t*) could be any function monotonically increasing with *t* where *z*_0_ = 0 and *z*_*T*_ = 1 (Schaal et al., 2003). For simplicity, we assumed a linear relationship between *t* and *z* in our analysis. Using these basis functions, one can effectively represent the trajectory τ in terms of a weight vector ω by resorting the position coordinates into two dimensions separately:

(7) $$\begin{array}{l} \tau =\left[\begin{array}{c} x_{1}\\ x_{2}\\ \vdots \\ x_{T}\\ y_{1}\\ y_{2}\\ \vdots \\ y_{T} \end{array}\right]=\left[\begin{array}{cc} \Phi_{1}^{†} & 0_{1\times n}\\ \Phi_{2}^{†} & 0_{1\times n}\\ \vdots & \\ \Phi_{T}^{†} & 0_{1\times n}\\ 0_{1\times n} & \Phi_{1}^{†}\\ 0_{1\times n} & \Phi_{2}^{†}\\ \vdots & \\ 0_{1\times n} & \Phi_{T}^{†} \end{array}\right]\;\left[\begin{array}{c} \omega_{x}\\ \omega_{y} \end{array}\right]+\epsilon_{\tau }=\Psi \omega +\epsilon_{\tau },\\ \;p(\tau |\omega )=N(\tau |\Psi \omega ,\Sigma_{\tau }) \end{array}$$

where ϵ_τ_~N(0, Σ_τ_) is zero-mean Gaussian noise. In our implementation we set $\Sigma_{\tau }=1e^{-10}I_{2T\times 2T}$ implying negligible observation noise of the trajectory.

To represent a distribution over multiple trajectory realizations of the same movement pattern we need to represent a distribution over weight vectors ω. We assume this distribution to be a Gaussian distribution *p*(ω|θ) = N(ω|μ_ω_, Σ_ω_) over the weight vector ω with summary statistics θ = {μ_ω_, Σ_ω_} composed of mean μ_ω_ and covariance Σ_ω_. Sample trajectories that follow a given pattern with statistics θ can be generated from the predictive distribution

(8) $$\begin{array}{l} p(\tau |\theta )=\int_{\omega }p(\tau |\omega )p(\omega |\theta )=\int_{\omega }N(\tau |\Psi \omega ,\Sigma_{q})N(\omega |\mu_{\omega },\Sigma_{\omega })\\ \;=N(\tau |\Psi \mu_{\omega },\Psi \Sigma_{\omega }\Psi^{†}+\Sigma_{\tau }) \end{array}$$

By taking the logarithm of the predictive distribution *p*(τ|θ) we get the log marginal likelihood that is crucial for Bayesian model complexity. As Equation (8) is Gaussian, the log marginal likelihood can be expressed analytically as

(9) $$\begin{array}{l} \log p(\tau |\theta )=-\underset{\text{goodness}-\text{of}-\text{fit}}{\underset{︸}{\frac{1}{2}{\left(\tau -\Psi \mu_{\omega }\right)}^{†}{\left(\Psi \Sigma_{\omega }\Psi^{†}+\Sigma_{\tau }\right)}^{-1}\left(\tau -\Psi \mu_{\omega }\right)}}\\ \;-\underset{\text{model complexity}}{\underset{︸}{\frac{1}{2}\log\det\left(\frac{\Psi \Sigma_{\omega }\Psi^{†}+\Sigma_{\tau }}{2\pi }\right)}} \end{array}$$

The data-independent model complexity corresponds to the entropy of the predictive distribution. This complexity is entirely determined by the covariance matrix Σ_ω_ that describes the variance of the movement pattern in feature space. We estimated the value of θ = {μ_ω_, Σ_ω_} in Equation (8) by Bayesian inference. To this end, we first computed the prior over θ by assuming a Normal-inverse-wishart distribution with parameters μ_0_, κ_0_, ν_0_, Σ_0_, and initialized μ_0_ and Ψ_0_ with maximum-likelihood estimates of mean and covariance over all data—see Lazaric and Ghavamzadeh (2010) for details of the maximum-likelihood estimation procedure. The parameter values κ_0_ and ν_0_ were initialized as κ_0_ = 0.1 and ν_0_ = 10.0. However, the correlation between complexity and subjective creativity judgment is robust and does not depend on the precise value of these parameters across several orders of magnitude (see Supplementary Figure 2). Given this prior over θ, the posterior over θ for each motion pattern also takes the form of a Normal-inverse-wishart with parameters [see for example Murphy (2012)]

$$\begin{array}{l} \;\mu =\frac{\kappa_{0}\mu_{0}+N\bar{x}}{\kappa_{0}+N}\\ \;\kappa =\kappa_{0}+N\\ \;\nu =\nu_{0}+N\\ \Psi =\Psi_{0}+\bar{\Psi }+\frac{\kappa_{0}N}{\kappa_{0}+N}\left(\bar{x}-\mu_{0}\right){\left(\bar{x}-\mu_{0}\right)}^{†}, \end{array}$$

where $\bar{x}$ is the sample mean, $\bar{\text{Ψ}}$ is the weighted sample covariance, and *N* is the number of observed trajectories for each pattern, i.e., *N* = 10. The values of {μ_ω_ = μ, Σ_ω_ = Ψ∕ν} provide a maximum-a-posteriori estimate of θ for a particular motion pattern obtained from subjects' example trajectories (10 required repetitions for each pattern). The main reason for computing maximum-a-posteriori estimates for the entropy is that maximum-likelihood estimates can lead to ill-conditioned covariance matrices (Murphy, 2012).

#### Symbolic sequence analysis

In a previous study (Peng et al., 2014) we have shown that motion complexity can be determined by translating continuous trajectories into symbol sequences consisting of up/down/left/right transitions. In order to obtain the transition sequence, we tessellated the workspace into unit squares called grid cells. In our experiment the workspace was 10 × 10 cm and for the analysis we used grid cell of 1 × 1 cm. Transitions between the grid cells are recorded as symbol sequences *s*_1_*s*_2_*s*_3_…*s*_*n*_, with *s*_*i*_ ∈ {*l, r, u, d*} corresponding to “left,” “right,” “up,” and “down.” To get reliable complexity estimates of such symbol sequences, we required at least 10, 000 transitions. As subjects only drew 10 trajectories per pattern, we fitted the distribution of Equation (8) to subjects' trajectories and sampled as many trajectories from this distribution as required to achieve at least 10, 000 transitions.

#### Lempel-Ziv complexity

Lempel-Ziv complexity is an irregularity measure for symbol sequences (Doğanaksoy and Göloğlu, 2006) that has also been widely applied in neuroscience (Radhakrishnan and Gangadhar, 1998; Blanc et al., 2008; Casali et al., 2013). Roughly, it counts the minimal number of distinct substrings to segment an entire symbol sequence. For instance, the decomposition of the binary sequence *x* = 01001101010111001001 into minimal blocks of the segmentation is 0|1|00|11|0101|0111|0010|01, hence the (LZ-76) complexity of *x* is 8.

#### Effective measure complexity

Effective measure complexity does not simply measure the degree of randomness, but rather the complexity of the structure of a sequence (Grassberger, 1986; Crutchfield and Feldman, 2003; Ay et al., 2006; Prokopenko et al., 2009). It is defined as

(10) $$EMC=\sum_{L=0}^{\infty }\left(h_{L}-h\right),$$

where the conditional entropy *h*_*L*_ quantifies the average uncertainty about the symbol *s*_*L*+1_ given the previous symbol sequence *s*_1_..*s*_*L*_. The longer the given sequence, the lower the conditional entropy, as adding more prior information can only lead to a better prediction of a symbol, such that *h*_*L*+1_ ≤ *h*_*L*_. The limit *L* → ∞ of the conditional entropy gives the *entropy rate* $h=\underset{L\to \infty }{\lim}h_{L}$, which provides a lower bound on all conditional entropies. We estimated the effective measure complexity for our sampled trajectories as described in Peng et al. (2014).

### Conflict of interest statement

The authors declare that the research was conducted in the absence of any commercial or financial relationships that could be construed as a potential conflict of interest.

## Footnotes

^1^http://socrates.berkeley.edu/~kihlstrm/RATest.htm
