# Supplementary material for: Entropic Movement Complexity Reflects Subjective Creativity Rankings of Visualized Hand Motion Trajectories
Source: Front Psychol. 2015 Dec 17;6:1879. doi: 10.3389/fpsyg.2015.01879 (PMC4681813; doi:10.3389/fpsyg.2015.01879)
Supplement: Supplementary file 1 [file DataSheet1.pdf]

Supplementary Material: Entropic movement complexity reflects subjective creativity rankings of visualized hand motion trajectories

Zhen Peng, Daniel A. Braun

*Correspondence:
Zhen Peng
zhen.peng@tuebingen.mpg.de

1 SUPPLEMENTARY FIGURE

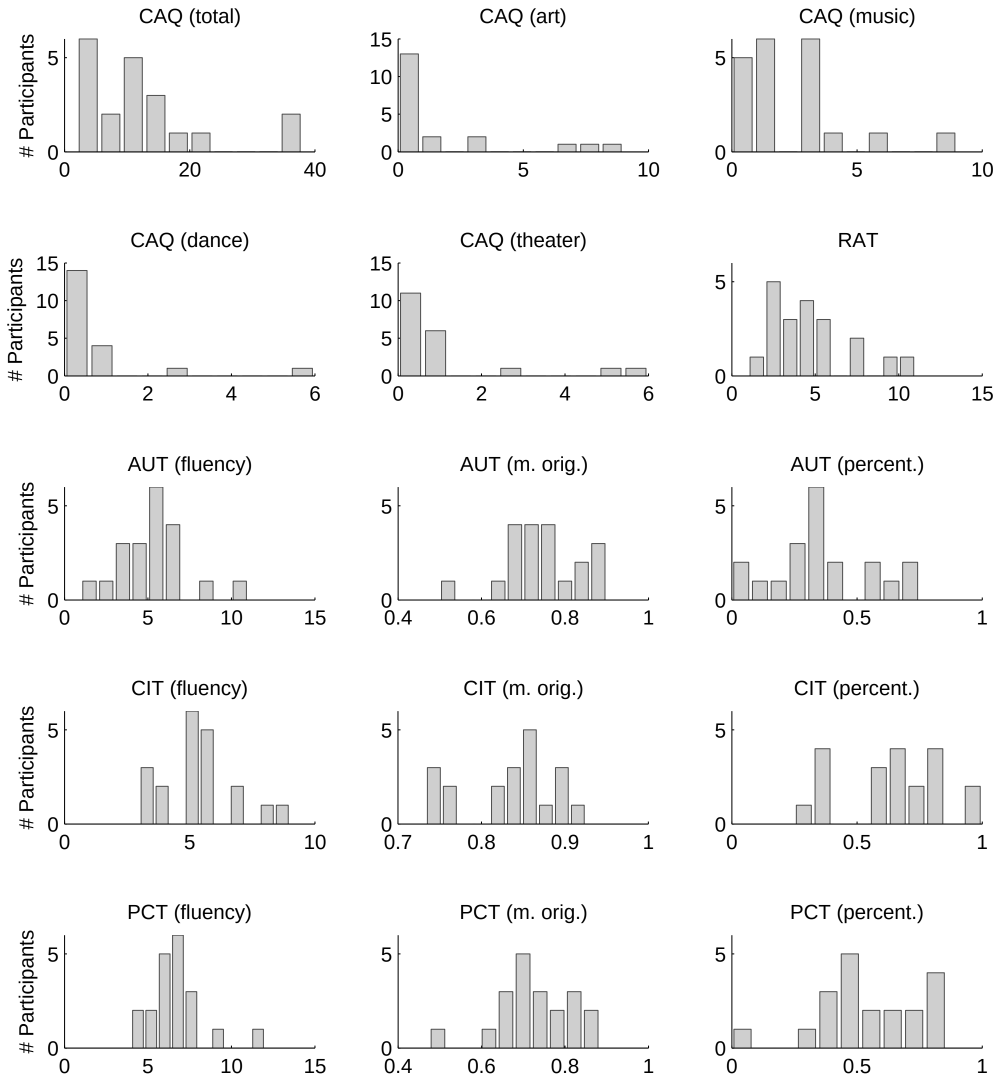

Supplementary Figure 1. Histogram of the scores archived by participants in the creativity tasks.

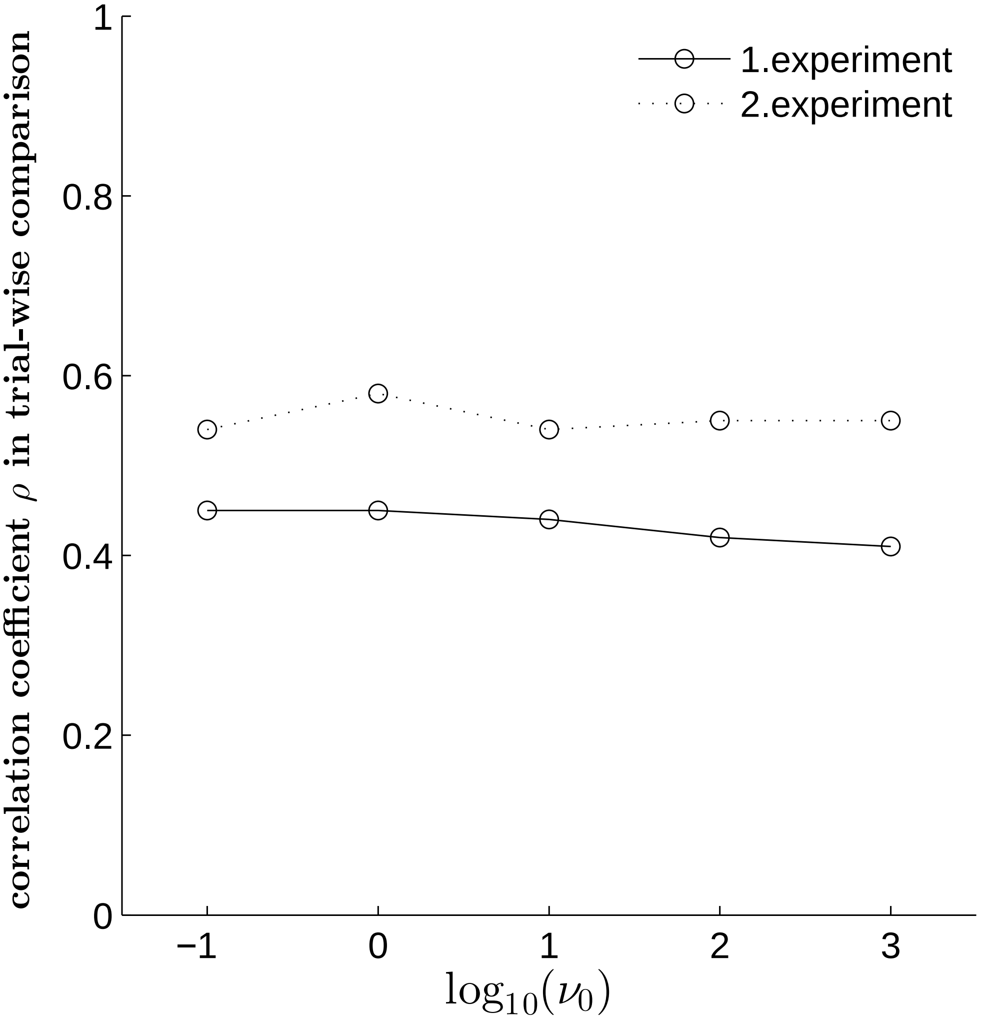

Supplementary Figure 2. Sensitivity analysis. Changing the parameter ν_0 does not have a strong impact on the correlation between computational complexity under the framework of Probabilistic Movement Primitives and subjective creativity rankings in trial-wise comparison. We tested five different settings of ν_0 across five orders of magnitude.
